# External radiotherapy combined with sorafenib has better efficacy in unresectable hepatocellular carcinoma: a systematic review and meta-analysis

**DOI:** 10.1007/s10238-022-00972-4

**Published:** 2022-12-10

**Authors:** Han Li, Zhenying Wu, Jiali Chen, Ke Su, Lu Guo, Ke Xu, Tao Gu, Yi Jiang, Pan Wang, Hao Zeng, Hao Chi, Kun He, Yunwei Han

**Affiliations:** 1 Department of Oncology, The Affiliated Hospital of Southwest Medical University, 25 TAIPING Street, Luzhou City, 646000 Sichuan Province China; 2 Department of Ophthalmology, The Affiliated Hospital of Southwest Medical University, 25 TAIPING Street, Luzhou City, 646000 Sichuan Province China; 3 Clinical Medical College, Southwest Medical University, Luzhou, 646000 China; 4 Clinical Research Institute, The Affiliated Hospital of Southwest Medical University, Luzhou, 646000 China

**Keywords:** Hepatocellular carcinoma, External radiotherapy, Adverse event, Sorafenib, Transarterial chemoembolization, Hepatic artery infusion chemotherapy

## Abstract

Advanced hepatocellular carcinoma (HCC) has a very low resectable rate. This meta-analysis aimed to compare efficacy of three combination strategies in treatment of advanced unresectable HCC with a view of guiding future selection of the best combination therapy for sorafenib and local therapy. A search was conducted to identify relevant literature published between April 2013 and May 2022, and then compared efficacy of sorafenib combined with external radiotherapy (SOF + RT), sorafenib with transarterial chemoembolization (SOF + TACE), sorafenib with hepatic artery infusion chemotherapy (SOF + HAIC), sorafenib (SOF), external radiotherapy (RT), transarterial chemoembolization (TACE), and hepatic artery infusion chemotherapy (HAIC) were studied and analyzed. Finally, the results were statistically analyzed using R 3.5.3 software and Stata/SE 15.0 software. A total of 46 studies, involving 7595 patients, were included in the meta-analysis. Analysis of overall survival (OS) and progression-free survival (PFS) of seven related treatment interventions revealed that the combination therapy had significantly higher efficacy than monotherapies. Among the combination therapies, SOF + RT was associated with the best OS and PFS rates, and the least adverse events compared to the other treatment modalities. The efficacy of combination therapy was better than monotherapy. In combination therapy, the overall survival time and progression-free survival time of SOF + RT were longer, and the adverse reactions were less. Therefore, SOF + RT may be the best choice for sorafenib combined with local therapy.

**Supplementary Information:**

The online version contains supplementary material available at 10.1007/s10238-022-00972-4.

## Introduction

Hepatocellular carcinoma (HCC) is the fourth most prevalent cancer in the world, with a 5-year survival rate of 18% [1]. The current guidelines for the diagnosis and treatment of primary HCC indicate that surgical resection remains the primary treatment option for early-stage HCC, while sorafenib (SOF) is the most widely used targeted drug for unresectable HCC [2, 3]. Studies have shown that some patients develop resistance to sorafenib which subsequently significantly affect patients’ OS. Therefore, combining sorafenib with other local treatments is imperative to improved efficacy [4, 5]. Local area therapies, such as external radiotherapy (RT), transarterial chemoembolization (TACE), and hepatic artery infusion chemotherapy (HAIC), have been associated with improved patient survival and quality of life [6–8]. Radiotherapy is increasingly used for the treatment of HCC [9, 10]. Consequently, some studies have demonstrated that sorafenib combined with external radiotherapy (SOF + RT) generates a synergistic effect which inhibits tumor growth [11, 12]. For instance, SOF + RT was associated with favorable OS and PFS with good tolerability, with a median OS of approximately 15.7 months for SOF + RT compared with a median OS of only 8.3 months for patients without local radiotherapy [13–16]. These findings indicate that a combination therapy of SOF + RT has great potential for treatment of HCC [17, 18].

Technological advancement has resulted in development and wide application of combination therapy comprising TACE and sorafenib (SOF + TACE). According to International Society of Multidisciplinary Interventional Oncology (ISMIO) consensus statement for 2021 [19], the use of TACE plus appropriate regimens can improve the outcome of unresectable HCC. Notably, patients in the SOF + TACE group were found to have significantly longer PFS rates than their counterparts in the TACE group (25.2 vs. 13.5 months, pendant 0.006). In recent years, numerous studies have reported that oxaliplatin- or cisplatin-based FOLFOX regimens applied to HAIC significantly improved tumor response rates and survival. For instance, results from a phase III trial [20] revealed that HAIC was associated with good OS rates and a manageable safety profile, with one study reporting a median overall survival time of 13.37 months for sorafenib with hepatic artery infusion chemotherapy (SOF + HAIC) (95%CI 10.27–16.46), a significant improvement compared to 7.13 months (95%CI 6.28–7.98) obtained when sorafenib was used alone [21–23]. Collectively, these findings indicate that SOF + HAIC has potential as an efficacious treatment modality for unresectable HCC.

Conversely, the use of sorafenib has been associated with a series of adverse events (AEs) that which may significantly affect patients’ quality of life [24–28]. Some of the most common AEs include fatigue, diarrhea, vomiting, loss of appetite, high blood pressure, and weight loss [28]. Appropriate strategies for management of AEs, coupled with use of sorafenib-based combination therapy, may help to bring better benefits to patients with advanced HCC [29]. The increase in first-line treatment for unresectable HCC represents a significant progress in the management of this malignant tumor. To date, however, data comparing efficacy of combination therapy based on sorafenib with other modalities are dearth, necessitating further research explorations that could guide selection of the most efficacious clinical treatment therapies. In this study, we systematically reviewed recent literature then performed a meta-analysis to compare efficacy of combination therapies and single therapies across clinical trials in treatment of HCC. Our findings are expected to guide future selection of the best treatment modality.

## Methods

This study followed the Preferred Reporting Items for Systematic Reviews and Meta-Analyses (PRISMA). We systematically searched PubMed, Embase, Medline, Web of Science, and Cochrane library databases for relevant literature targeting randomized controlled trials and observational studies published from April 2013 to May 2022. The keywords used in the search included carcinomas, hepatocellular, liver neoplasms, transarterial chemoembolization, external radiotherapy, targeted radiotherapies, hepatic artery infusion chemotherapy, and sorafenib. Next, we read the title and abstract of each retrieved article and excluded all irrelevant researches, referred to the full text of the articles involved, and strictly controlled the inclusion criteria and exclusion criteria.

### Selection criteria

Articles were included in this meta-analysis if they met the following criteria: (1) study design: randomized controlled trials or observational comparative studies; (2) population: patients with HCC did not meet the criteria of surgical resection and were staged in middle and late stages; and (3) intervention: patients were treated with SOF + RT, SOF + TACE, SOF + HAIC, TACE, RT, HAIC, or SOF therapies. On the other hand, studies that met the following criteria were excluded from the analysis: (1) conference abstracts, review articles, case reports, non-control studies; (2) study population included patients with early-stage HCC, diffuse HCC, tumor diameter < 3 cm; (3) lacked adequate data; and (4) single-arm studies containing the above treatments.

### Data extraction

The following information was extracted from each article: (1) article information: first author’s last name, year of publication, intervention, and sample size; (2) patient-related information: Barcelona Clinic Liver Cancer (BCLC) or American Joint Committee on Cancer (AJCC) stage, alpha-fetoprotein (AFP) level, Child–Pugh grade, as well as tumor size. (3) data: survival time (OS), progression-free survival time (PFS), Kaplan–Meier curve, adverse reactions (AEs), risk ratio (HR), confidence interval (CI) of 95% different interventions, chart information in the article, complications, factors affecting patient survival, AEs, as well as 1-, 2-, and 3-year survival rates. The main results targeted in this meta-analysis included OS (based on HR), PFS (based on HR), and AEs. And we used (GetdataGraphDigitizer2.26) to extract the studies in which survival rates could not be obtained.

### Definitions

OS was defined as the time from the date of treatment to the patient death, PFS was defined as progression-free survival time, HR is defined as the risk ratio of the control group to the intervention group, and AEs are defined as the number of patients with adverse reactions after treatment.

### Study characteristics

IT was retrieved from April 1, 2022, to May 2022. After repeated elimination and preliminary screening, 46 studies [13, 20–23, 30–64], including 7595 patients with unresectable HCC, were subjected to data extraction and analysis. Identification, screening, and inclusion of studies were performed in accordance with PRISMA guidelines. A summary of articles included in this meta-analysis is provided in Table 1. Thirty-three of the included studies were retrospective studies, with a 13 of prospective ones.

**Table 1:** Baseline characteristics

Author	Year	Design	Arm	No	BCLC(B/C) or AJCC(III/IV)	Child (A/B)	AFP ≤ 400/ > 400 ng/L	Tumor size (cm)	NOS score
Liu	2021	Retrospective	TACE	48	15/33	35/13	20/28	11.0 ± 3.0	7
			SOF + TACE	42	13/29	31/11	18/24	11.5 ± 3.7	
Yuan	2019	Retrospective	TACE	138	0/138	133/5	NA	8.55 ± 3.39	8
			SOF + TACE	69	0/69	67/2	NA	8.39 ± 4.45	
Lencioni	2016	Prospective	TACE	153	NA/NA	152/0	112/41	NA	7
			SOF + TACE	154	NA/NA	153/1	113/41	NA	
Kudo	2020	Prospective	TACE	76	34/9	71/5	NA	NA	8
			SOF + TACE	80	44/9	79/1	NA	NA	
Ren	2019	Retrospective	TACE	122	72/50	111/11	77/45	> 5 (77)	6
			SOF + TACE	61	30/31	55/6	42/19	> 5 (35)	
Zhang	2016	Retrospective	TACE	60	NA/NA	NA/NA	18/42	10.3 ± 3.4	7
			SOF + TACE	20	NA/NA	NA/NA	7/13	9.6 ± 4.0	
Liu	2020	Retrospective	TACE	40	0/40	24/16	21/19	6.9 (1.6–12.0)	6
			SOF + TACE	35	0/35	23/12	12/23	7.4 (2.1–11.7)	
Bai	2013	Prospective	TACE	164	45/115	115/49	NA	NA	6
			SOF + TACE	82	19/63	63/19	NA	NA	
Koch	2021	Retrospective	SOF	82	0/82	61/21	52/30	NA	8
			TACE	65	0/65	50/15	40/25	NA	
			SOF + TACE	54	0/54	40/14	36/18	NA	
Peng	2021	Retrospective	TACE	112	19/53	41/47	67/45	> 5 (84)	8
			SOF + TACE	56	16/27	39/14	37/19	> 5 (34)	
Wan	2016	Retrospective	TACE	245	NA/NA	218/27	170/75	< 5 (99)	7
			SOF + TACE	245	NA/NA	213/32	132/113	< 5 (99)	
Kaibori	2021	Retrospective	TACE	29	29/0	29/0	NA	1.4 (0.6–3.3)	8
			SOF + TACE	41	41/0	41/0	NA	2.1 (0.7–8.1)	
Hu	2014	Retrospective	TACE	164	0/164	103/63	45/119	NA	7
			SOF + TACE	82	0/84	58/24	29/55	NA	
Meyer	2017	Prospective	TACE	156	NA/NA	148/3	NA	5 (4–8)	9
			SOF + TACE	157	NA/NA	145/5	NA	6 (4–8)	
Wu	2017	Retrospective	SOF	56	10/46	45/11	33/23	9.1 (1–19.5)	6
			SOF + TACE	48	16/32	46/2	23/24	7.65 (1–19.0)	
Su	2021	Prospective	SOF	24	10/12	17/7	NA	NA	5
			SOF + TACE	18	9/9	14/4	NA	NA	
Lee	2020	Retrospective	SOF	65	NA/NA	NA/NA	NA	> 5 (31)	7
			SOF + TACE	53	NA/NA	NA/NA	NA	> 5 (15)	
Zhang	2015	Retrospective	SOF	44	30/14	34/10	NA	NA	7
			SOF + TACE	45	32/13	34/11	NA	NA	
Park	2018	Prospective	SOF	169	44/125	147/22	NA	NA	6
			SOF + TACE	170	39/128	148/22	NA	NA	
Zhao	2020	Retrospective	SOF	90	0/90	83/7	43/47	10.0 (7.1–11.9)	7
			TACE	233	0/233	214/19	128/15	8.7 (6.4–11.8)	
Kirstein	2017	Retrospective	SOF	98	NA/NA	NA/NA	NA	NA	7
			TACE	73	NA/NA	NA/NA	NA	NA	
Liu	2021	Retrospective	RT	73	64/7	68/5	NA	> 10 (28)	8
			SOF + RT	73	65/6	69/4	NA	> 10 (28)	
Abulimiti	2021	Retrospective	RT	46	29/17	44/2	24/22	< 10 (30)	7
			SOF + RT	36	18/18	35/1	17/19	< 10 (38)	
Que	2019	Retrospective	RT	36	NA/NA	31/5	20/16	< 10 (20)	7
			SOF + RT	18	16/2	15/3	10/8	< 10 (16)	
Yoshiyuki	2018	Retrospective	RT	47	0/47	47/0	NA	NA	6
			SOF + RT	15	0/15	15/0	NA	NA	
Sun	2016	Retrospective	RT	22	NA/NA	NA/NA	NA	NA	8
			SOF	18	NA/NA	NA/NA	NA	NA	
			SOF + RT	23	NA/NA	NA/NA	NA	NA	
Chang	2022	Retrospective	SOF	330	0/330	299/31	181/149	NA	8
			SOF + RT	68	0/68	60/8	47/21	NA	
Bettinger	2018	Prospective	SOF	95	42/48	70/25	NA	6.5 ± 4.1	7
			RT	95	48/43	67/28	NA	6.2 ± 3.6	
Nakazawa	2014	Retrospective	SOF	28	NA/NA	NA/NA	NA	NA	8
			RT	28	NA/NA	NA/NA	NA	NA	
Liang	2021	Retrospective	HAIC	126	0/126	126/0	39/87	NA	7
			SOF + HAIC	99	0/99	99/0	25/74	NA	
Miyaki	2019	Retrospective	HAIC	164	41/118	133/31	NA	NA	8
			SOF + HAIC	27	9/16	25/2	NA	NA	
He	2019	Prospective	SOF	122	0/122	NA/NA	NA	NA	8
			SOF + HAIC	125	0/125	NA/NA	NA	NA	
Kudo	2018	Prospective	SOF	103	27/76	93/10	57/46	NA	8
			SOF + HAIC	102	32/70	90/12	46/49	NA	
Kondo	2019	Prospective	SOF	33	13/18	29/4	NA	NA	7
			SOF + HAIC	35	14/19	31/4	NA	NA	
Zheng	2022	Prospective	SOF	32	0/32	27/5	NA	10.7 ± 3.9	7
			SOF + HAIC	32	0/32	28/4	NA	10.6 ± 4.0	
Ikeda	2016	Prospective	SOF	41	16/25	39/2	NA	5.2 (1.1–17.5)	8
			SOF + HAIC	66	19/46	57/8	NA	5.1 (1–20)	
Nagai	2015	Retrospective	HAIC	20	0/20	6/14	NA	NA	8
			SOF + HAIC	18	0/18	11/7	NA	NA	
Kotaro	2015	Retrospective	SOF	72	NA/NA	61/11	NA	< 5 (44)	7
			HAIC	128	NA/NA	79/49	NA	< 5 (62)	
Lyu	2018	Retrospective	SOF	232	NA/NA	NA/NA	NA	NA	5
			HAIC	180	NA/NA	NA/NA	NA	NA	
Zaizen	2021	Retrospective	SOF	83	74/9	59/24	NA	NA	5
			HAIC	83	71/12	53/30	NA	NA	
Aoka	2015	Retrospective	SOF	41	3/38	NA/NA	NA	4 (1–19)	8
			HAIC	136	1/135	NA/NA	NA	4 (1–18)	
Choi	2018	Prospective	SOF	29	0/29	25/4	NA	> 10 (17)	8
			HAIC	29	0/29	27/2	NA	> 10 (15)	
Kang	2018	Retrospective	SOF	44	17/25	NA/NA	NA	7.4 ± 3.5	7
			HAIC	95	19/72	NA/NA	NA	8.1 ± 3.7	
Ahn	2020	Retrospective	SOF	35	0/35	24/11	NA	NA	6
			HAIC	38	0/38	27/11	NA	NA	
Moriguchi	2017	Retrospective	SOF	14	0/14	14/0	NA	6.58 (3.27–10.8)	8
			HAIC	32	0/32	32/0	NA	7.47 (0–17.91)	
Song	2015	Retrospective	SOF	60	0/60	47/13	NA	> 10 (29)	4
			HAIC	50	0/50	45/5	NA	> 10 (28)	

### Data screening and quality evaluation

The two researchers independently read the titles and abstracts of the retrieved articles and then selected according to the aforementioned inclusion criteria. Next, they read each article’s full text and finally selected those that met our inclusion criteria. A single table was prepared and used to extract the following information: the name of the author and title, interventions, Barcelona Clinic Liver Cancer, Child–Pugh grading, tumor diameter, important outcome indicators, and research quality indices. In most studies, Response Evaluation Criteria in Solid Tumors (RECIST), modified RECIST or World Health Organization (WHO) criteria were used to evaluate the efficacy. Most studies adopted Common Terminology Criteria for Adverse Events (CTCAE) proposed by the radiotherapy Oncology Group (RTOG) and the European Organization for Cancer Research and treatment (EORTC) [65–67]. Since most of the included studies were of a retrospective design, we used the Nottingham Ottawa scale (NOS) to assess the quality of each study and found that most of the studies were of medium quality. The two researchers independently rated and discussed until the results were consistent. The articles were stratified according to NOS scores, with 7–9 and 4–6 denoting high-quality and medium-quality reports, respectively, and those with scores below 4 classified as low quality.

### Statistical analysis

Primary observation endpoints were OS, PFS, and AEs, and hazard ratio (HR) was used to compare OS and PFS. After adjustment, we applied the following specific algorithm for indirect comparison: Ln (HR) = [ln (UL − HR) + ln (LL − HR)]/2 UL − HR seln (HR) = [ln (UL − HR) − ln (LL − HR)]/(1.96 × 2). Stata/SE 15.0 software and R 3.5.3 software were used for meta-analysis and then used SUCRA score of survival to discuss the ranking probability. We also used network meta-analysis to synthesize the included information then applied frequency distribution for direct and indirect comparisons. Publication bias was analyzed using Funnel chart and Egger’s test. To this end, a symmetrical graphic representation indicated no obvious publication bias, while an asymmetric profile denoted publication bias. All statistical analyses were performed at a significance level of *p* < 0.05.

## Results

### Included studies

**Fig. 1:**
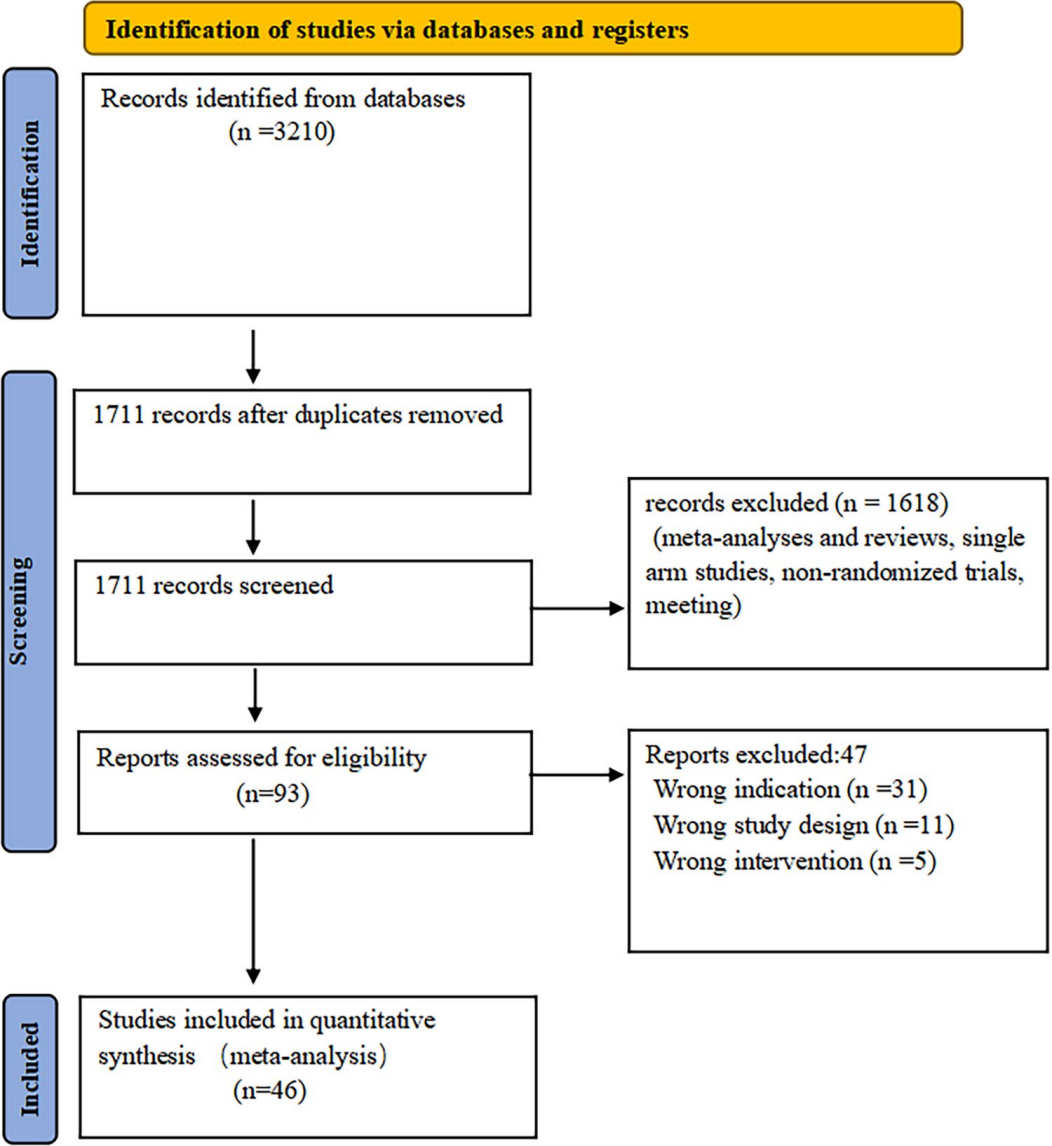
PRISMA flowchart

Initial literature search yielded a total of 3210 articles, of which 1711 were deleted on the account of being duplicates. After the screening of literature types, 1618 articles were excluded. Further full-text review resulted in deletion of 47 articles. Finally, 46 studies met our inclusion criteria and were therefore included in the meta-analysis. Table 1 summarizes the general characteristics of the included studies. And the selection process is illustrated in Fig. 1.

### Study characteristics

**Fig. 2:**
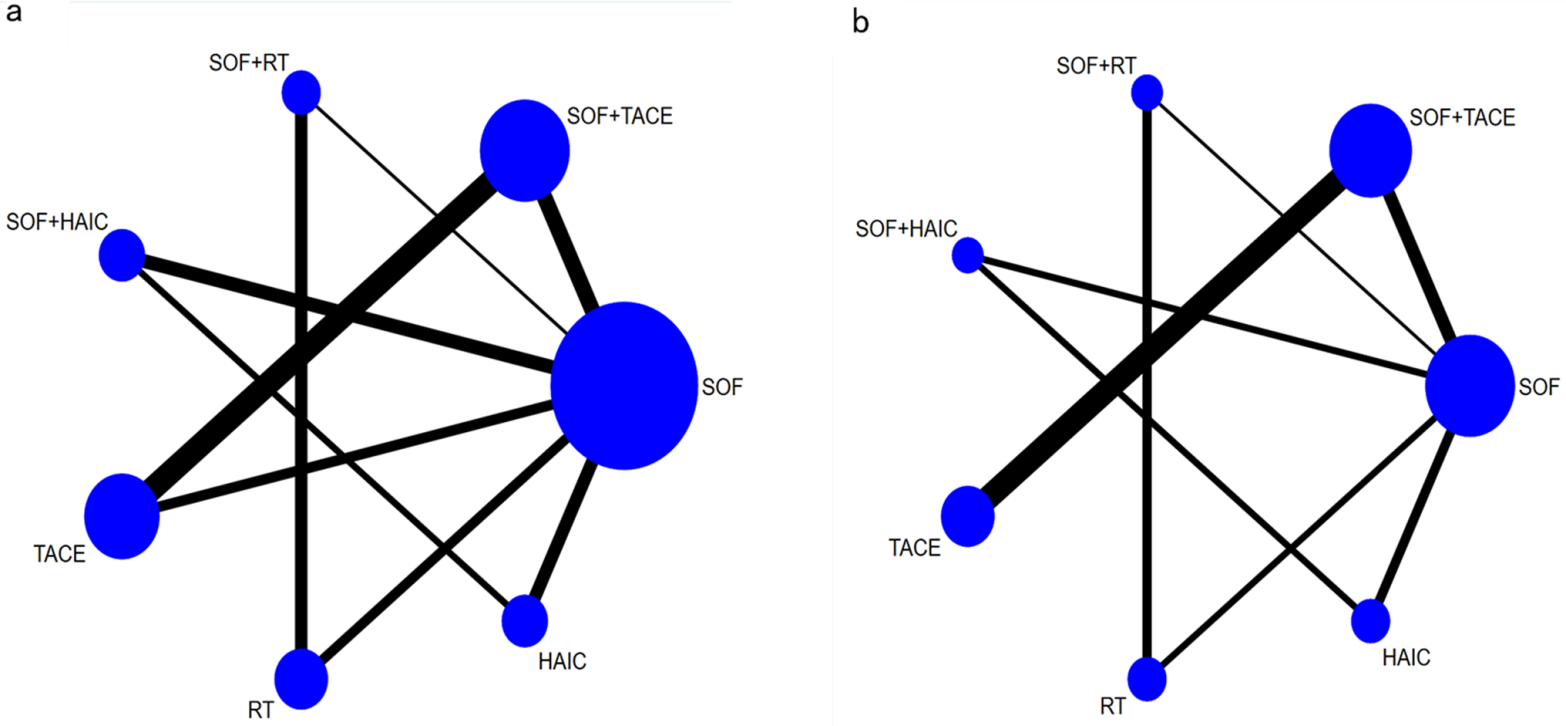
Networks of OS **a** and AEs **b** reported in included studies

All the clinical studies included were double-arm randomized controlled trials, and all described patients with unresectable HCC. Network analysis results for OS and AEs are shown in Fig. 2, respectively. In the current study, we compared 7 different combination and non-combination treatments, namely SOF, SOF + TACE, SOF + RT, SOF + HAIC, TACE, RT, and HAIC, with the aim of identifying the best combination of sorafenib plus local therapy. The mesh map shows a direct comparison between different treatment arms. The size of each circle represents the number of relevant literatures included in this treatment, and the number of head-to-head comparisons between adjacent pre-arms is proportional to the thickness of the connection line. In the specific strategy of external radiotherapy, most of the studies used stereotactic body radiotherapy (SBRT), and only 4 studies used intensity modulated radiotherapy (IMRT) or 3-dimensional conformal radiotherapy (3D-CRT). We conducted a subgroup analysis and found that there was no significant difference in the effect of different types of radiotherapy on the efficacy.

### Overall survival

**Fig. 3:**
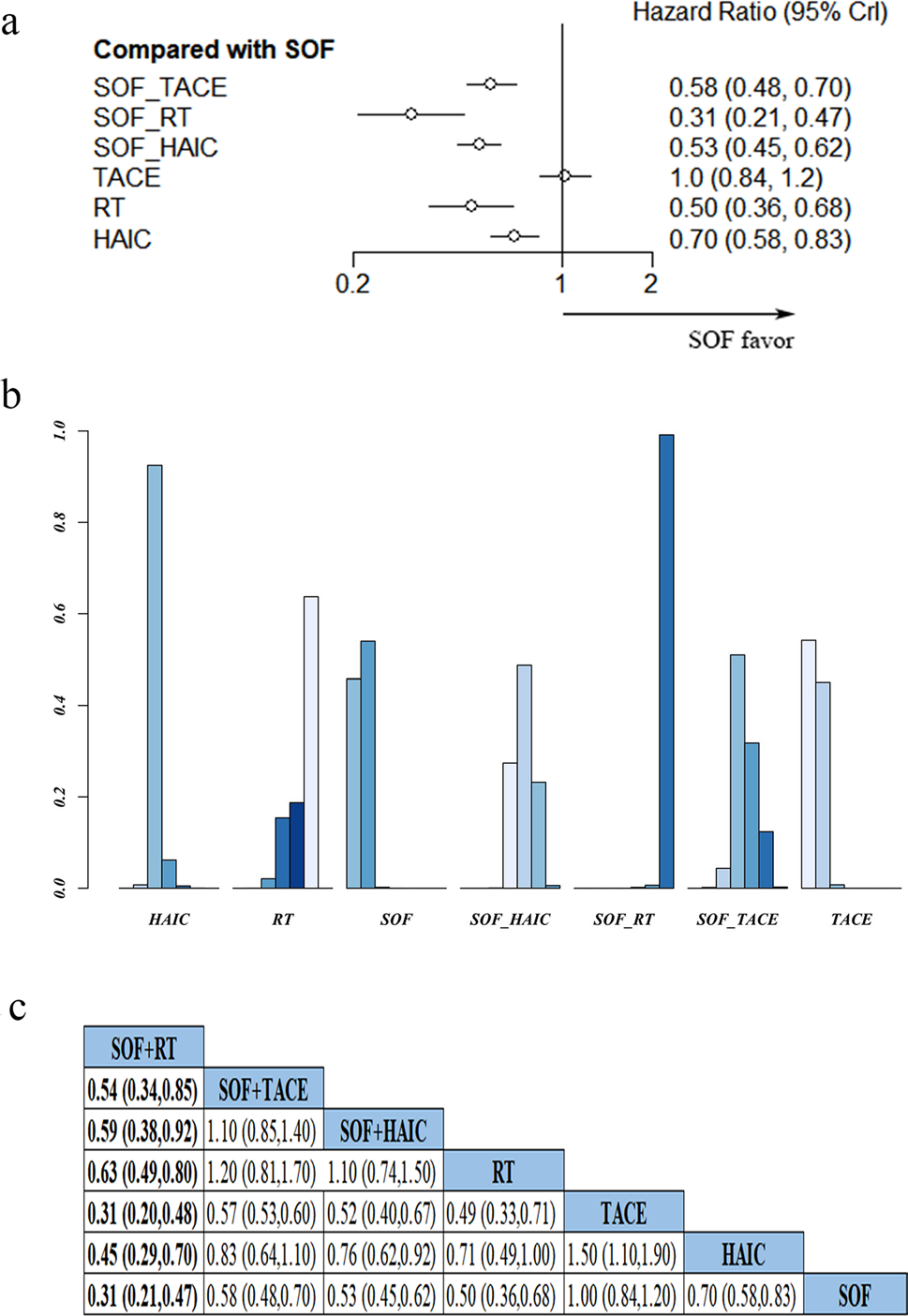
**a** Forest plots showing pairwise comparison of OS across various treatment methods. **b** Probability histogram of OS in various treatments; **c** HRs depicting pairwise comparisons of OS between treatments

A total of 43 studies reported OS. Regarding specific survival rates, 42, 39 and 24 studies reported 1-, 2-, and 3-year survival rates, respectively, as shown in Supplementary Table 1. In addition, 7 interventions were reported, although we found no statistically significant heterogeneity among the studies. To further explore efficacy levels of these treatments, we generated forest maps (Fig. 3a) and cumulative probability histograms (Fig. 3b) by measuring the HR and 95%CI of OS in the experimental relative to control groups across 36 studies. Forest plots showed that the combination therapy had significantly better efficacy than monotherapy (SOF + RT > SOF/RT, SOF + TACE > SOF/TACE, SOF + HAIC > SOF/HAIC) for treatment of unresectable HCC (Fig. 3a). SOF + TACE, SOF + RT, and SOF + HAIC were also associated with improved OS of patients compared to sorafenib alone (HR and 95%CI 0.58, 0.48–0.70; 0.31, 0.21–0.47; 0.53, 0.45–0.62). Results from sorafenib with local therapy revealed that SOF + RT > SOF + HAIC > SOF + TACE, suggesting that SOF + RT may be the best choice for the benefit of OS. Results from network pairwise comparison of seven studies showed that SOF + RT achieved significant benefits on OS (Fig. 3c).

### Progression-free survival

**Fig. 4:**
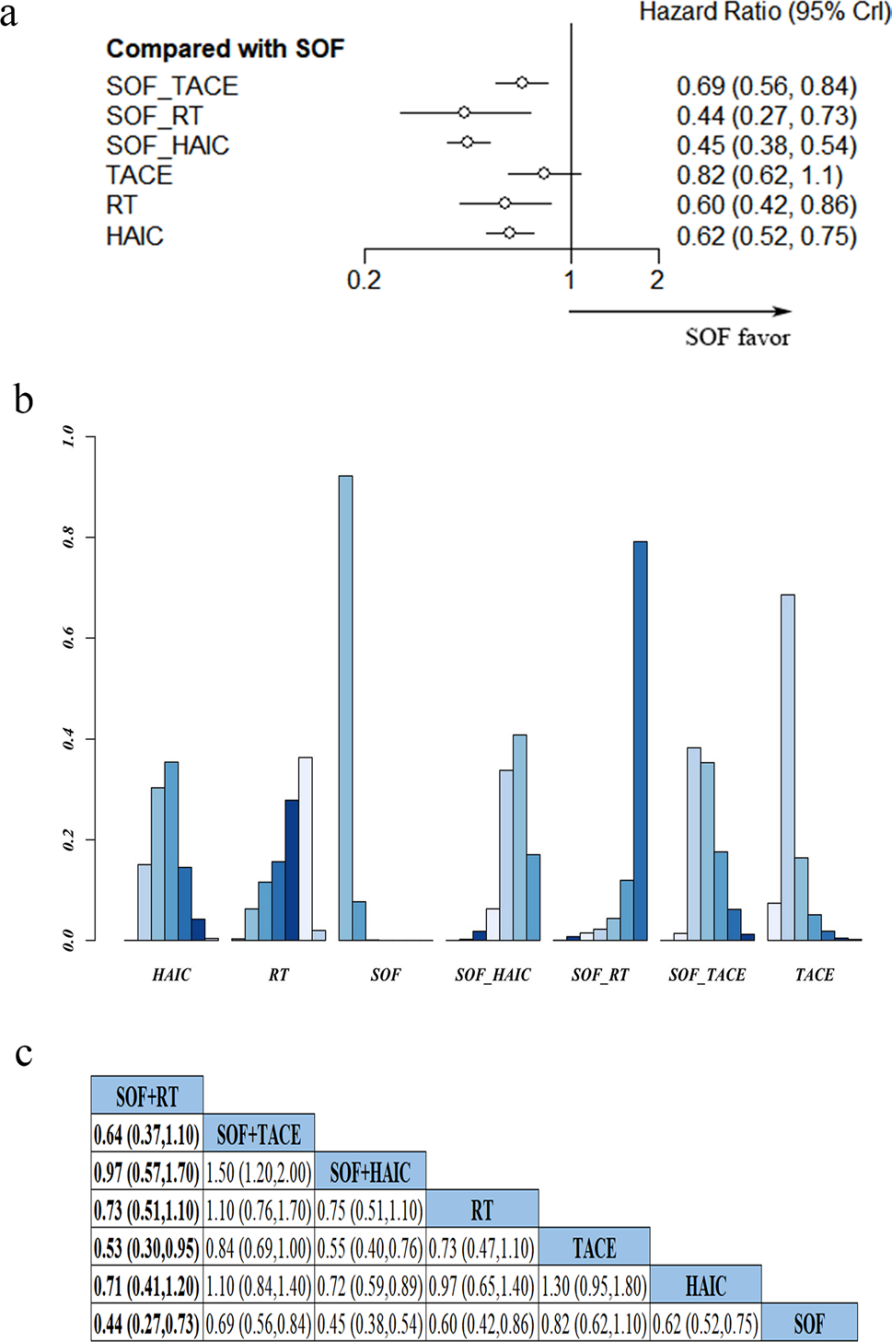
**a** Forest map showing pairwise comparisons in patients PFS across various treatment therapies. **b** Probability histogram of PFS in various treatments; **c** HRs depicting pairwise comparisons of PFS between treatments

Next, we generated forest maps (Fig. 4a) and cumulative probability histogram (Fig. 4b) from 15 studies in order to compare effect of various intervention therapies on patients’ PFS by measuring the HR and 95%CI of their PFS. Results showed that patients treated with SOF + TACE, SOF + RT, and SOF + HAIC had better PFS than those treated with sorafenib monotherapy (HR and 95%CI 0.69, 0.56–0.84; 0.44, 0.27–0.73; 0.45, 0.38–0.54). Among the combination therapies, SOF + RT was associated with the best PFS. Meanwhile, analysis of the combination between sorafenib and local therapy revealed benefit to PFS is in the following order: SOF + RT > SOF + HAIC > SOF + TACE. A network of pairwise comparison among seven studies showed that SOF + RT achieved the best benefits on patients’ PFS (Fig. 4c).

### Adverse events

**Fig. 5:**
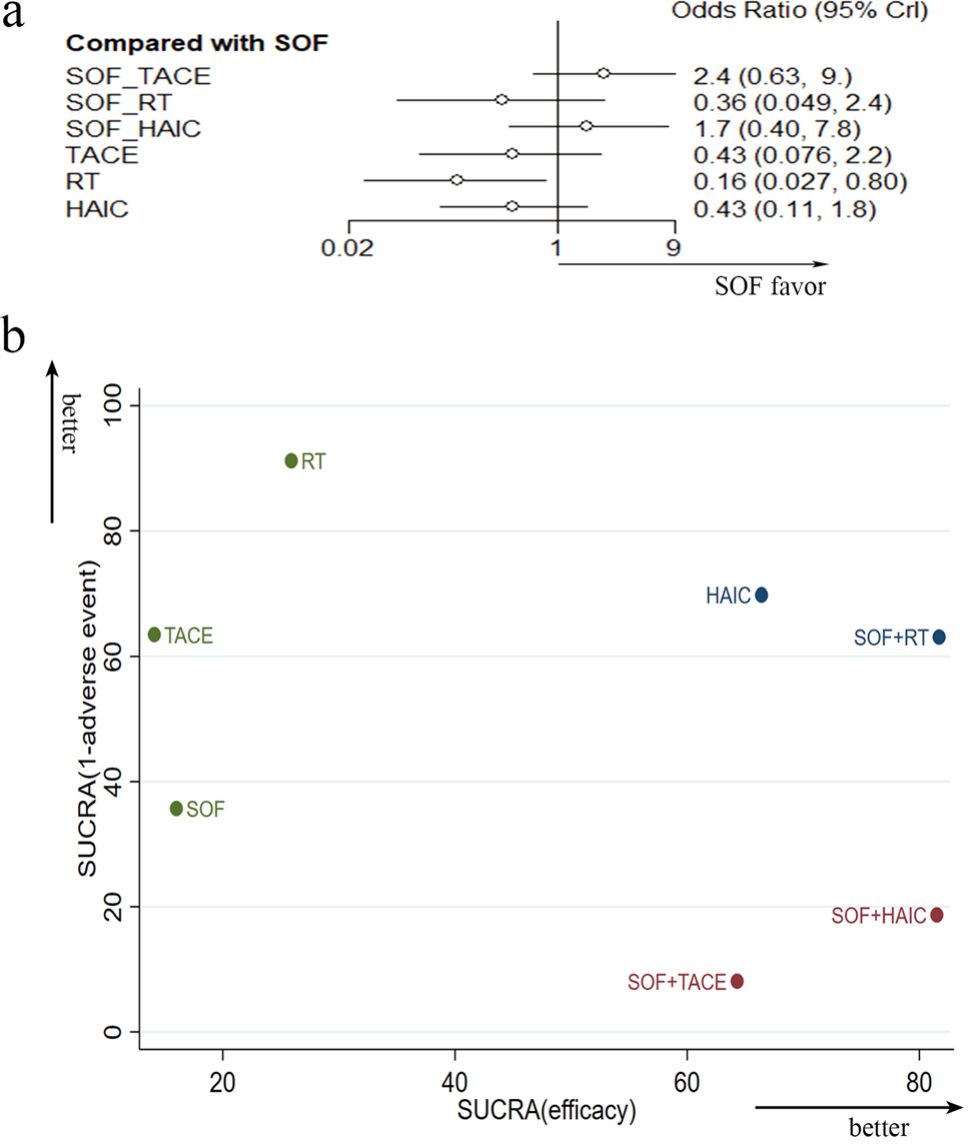
**a** Forest map showing pairwise comparison of AEs associated with various treatment methods. **b** Clustered ranking plot of the acute mania network based on cluster analysis two different outcomes: survival (SUCRA efficacy) and tolerability (SUCRA 1-AEs)

A total of 24 studies reported grade3/4 grade AEs, which were subsequently extracted for generation of a forest map (Fig. 5a). Summarily, results indicated that RT, TACE, and HAIC alone were associated with the least AEs, while sorafenib alone or sorafenib combined with local treatment caused numerous AEs. Among the combination therapies, SOF + RT was associated with relatively lower AE occurrence. Moreover, we calculated the SUCRA score for survival and AEs. According to the SUCRA values of survival (efficacy) and tolerability (1-adverse event), we made a clustered ranking plot to rank them and found that SOF + RT elicited relatively fewer AEs and considerable clinical benefits in combination therapy (Fig. 5b).

### Publication bias

We use the funnel diagram to assess the potential bias risk of meta-analysis, and the funnel diagram is roughly symmetrical, which proves that our conclusion is unlikely to be wrong.

## Discussion

In this study, we provide the first meta-analysis describing efficacy of sorafenib combined with local therapy. Although numerous studies have associated local therapy combined with TKIs with favorable OS, direct comparison among these treatment combinations remains a challenge. In this meta-analysis, we included a large number of relevant studies to compare efficacy of these combinations on HCC patients through OS rankings calculated by risk ratio HR. Our results indicated that the efficacy of combined therapy is significantly better than that of monotherapy. In addition, SOF + RT was associated with better OS, PFS, and fewer adverse reactions compared to the three combination therapies. These findings are expected to guide doctors during future decision making on the best treatment modality for HCC. Previous studies have shown that external radiotherapy can play a crucial role in management of HCC [68–71]. Notably, researchers have recommended that more focus should be directed to the importance of radiotherapy and radiotherapy combined with targeted therapy during treatment of unresectable HCC.

Sorafenib was approved for advanced HCC based on the results of the SHARP trial and subsequently confirmed by another Asia–Pacific trial [72–74]. Results from previous retrospective and prospective trials have shown that RT can achieve effective clinical benefits and low risk of self-injury. For instance, Huang et al. [75] demonstrated that sorafenib not only sensitized drug-resistant cancer cells but also induced radiation-induced apoptosis by downregulating STAT3 phosphorylation. On the other hand, Su et al. [76, 77] showed that RT was more efficacious than TACE for treatment of advanced HCC, possibly because obstruction of the portal vein in advanced HCC lowers its therapeutic effect. Results from another study indicated that sorafenib was associated with improved radiosensitivity while its combination therapy caused a significant delay in tumor growth [78]. Moreover, TACTIC test results showed that SOF + TACE had a positive therapeutic effect than TACE monotherapy for treatment of unresectable HCC [33]. On the other hand, local treatment was found to induce release of antigens and pro-inflammatory cytokines, cooperate with tyrosine kinase inhibitors to enhance immunity, and inhibit primary tumor checkpoint [79]. Previous studies have also shown that sorafenib is an effective poly-kinase inhibitor that targets the vascular endothelial growth factor (VEGF) receptor, and therefore, it is expected to have a synergistic effect when combined with TACE in the treatment of HCC [2]. Data from a recent Phase III FOHAIC-1 trial revealed the efficacy of hepatic arterial infusion of fluorouracil, calcium folinate, and oxaliplatin (HAIC-FO) for treatment of advanced HCC. Notably, patients with advanced HCC who were treated with HAIC exhibited better survival times than their counterparts who received sorafenib, although it was associated with a high burden of intrahepatic disease. In addition, HAIC significantly improved the overall survival of patients with unresectable large HCC compared to TACE. SOF + HAIC was also associated with better overall survival and acceptable toxicity rates in HCC patients than sorafenib [21].

Results from the present meta-analysis are expected to guide future selection of systematic combined with local therapy for treatment of advanced unresectable HCC. Various treatment therapies have been associated with adverse reactions. Numerous studies have shown that sorafenib monotherapy can result in several adverse reactions, possibly due heterogeneity in the drug’s pharmacokinetics in HCC patients. Studies have also shown that oncogenes tend to be hypermethylated, whereas tumor suppressor genes tend to be hypomethylated after sorafenib treatment, indicating that sorafenib can affect methylation levels of genes regulating cancer development and progression as well as the related pathways in HCC cells, thereby causing serious side effects [80, 81]. Although the combination therapy has many benefits, it is also associated with numerous adverse reactions, such as diarrhea, vomiting, hypertension, hand and foot adverse reactions, fatigue, and fever. However, almost all patients can tolerate it, with studies confirming safety of sorafenib and various local combinations. At the same time, studies have shown that the adverse reactions caused by SOF + RT are relatively few in the combination therapy, although the choice of a specific treatment still depends on a patient’s basic physical conditions. Therefore, clinical choice of a treatment modality should be based on each patient’s basic situation, coupled with comprehensive understanding of efficacy and the associated adverse reactions.

This study had some limitations. First of all, many of the studies we have included are retrospective studies, so there is always a risk of publication bias. Secondly, heterogeneity of age, sex, AFP level, and ECOG score may lead to inevitable bias, so more subgroup analysis should be done. Finally, the selection bias in the included literature is also a major factor affecting the quality of the article.

Targeted therapy cannot produce a complete and lasting tumor response, which eventually leads to drug resistance and tumor recurrence [82]. In addition to the combination therapy based on targeted therapy, various combination therapies based on immunotherapy also prolong OS and have controllable safety, such as atezolizumab plus bevacizumab, camrelizumab plus apatinib, and pembrolizumab plus lenvatinib. Future studies are expected to explore efficacy of combining pd-1 with other treatments.

And lenvatinib, which is also a targeted drug, has shown excellent efficacy in treatment of HCC. Notably, lenvatinib acts on radiation-induced Src/STAT3/NF-κB signal transduction to enhance the antitumor effect of radiation on hepatocellular carcinoma. Previous studies have shown that lenvatinib not only promotes radiation effect of hepatocellular carcinoma cell proliferation on inhibition, inhibition of invasion, and induction of apoptosis, but also reduces radiation-triggered carcinogenesis and EMT (epithelial–mesenchymal transition)-related protein expression [83]. Therefore, lenvatinib combined with external radiotherapy may also have a good effect.

And lenvatinib-TACE sequential therapy also showed deep remission and good prognosis, indicating that adding TACE to lenvatinib can improve the clinical outcome, and thus, it is a potential treatment choice for patients with advanced hepatocellular carcinoma. Efficacy of the combination therapy needs to be validated using prospective and retrospective studies with large sample sizes. Even some studies have also shown that TACE in combination with lenvatinib has excellent efficacy than TACE combined with sorafenib for treatment of advanced hepatocellular carcinoma. However, due to the limited study and sample size of lenvatinib plus local therapy, and based on the rich sample size of this study, we still use sorafenib as indirect evidence for the horizontal comparison of systemic therapy and local therapy. Further research exploration is needed to verify efficacy of both targeted and local therapies [84–89].

## Conclusion

The efficacy of combination therapy was better than monotherapy. In combination therapy, the median survival time and progression-free survival time of SOF + RT were longer, and the adverse reactions were less. Therefore, SOF + RT may be the best choice for sorafenib combined with local therapy.

## Supplementary Information

Below is the link to the electronic supplementary material.

Supplementary file1 (DOCX 33 kb)

## Abbreviations

HCC: Hepatocellular carcinoma
OS: Overall survival
PFS: Progression-free survival
RT: External radiotherapy
TACE: Transarterial arterial chemoembolization
HAIC: Hepatic artery infusion chemotherapy
BCLC: Barcelona clinic liver cancer
AJCC: American Joint Committee on Cancer

## References

[1] Llovet JM, Kelley RK, Villanueva A, Singal AG, Pikarsky E, Roayaie S (2021). Hepatocellular carcinoma. Nat Rev Dis Prim.

[2] Couri T, Pillai A (2019). Goals and targets for personalized therapy for HCC. Hepatol Int.

[3] Choi C, Choi GH, Kim TH, Tanaka M, Meng MB, Seong J (2014). Multimodality management for Barcelona clinic liver cancer stage C hepatocellular carcinoma. Liver Cancer.

[4] Dhir M, Melin AA, Douaiher J, Lin C, Zhen WK, Hussain SM (2016). A Review and update of treatment options and controversies in the management of hepatocellular carcinoma. Ann Surg.

[5] Ahmed O, Pillai A (2020). Hepatocellular carcinoma: a contemporary approach to locoregional therapy. Am J Gastroenterol.

[6] Chapiro J, Duran R, Geschwind JF (2014). Combination of intra-arterial therapies and sorafenib: is there a clinical benefit?. Radiol Med.

[7] Raoul JL, Forner A, Bolondi L, Cheung TT, Kloeckner R, de Baere T (2019). Updated use of TACE for hepatocellular carcinoma treatment: how and when to use it based on clinical evidence. Cancer Treat Rev.

[8] Ogasawara S, Koroki K, Kanzaki H, Kobayashi K, Kiyono S, Nakamura M (2022). Changes in therapeutic options for hepatocellular carcinoma in Asia. Liver Int.

[9] Bujold A, Massey CA, Kim JJ, Brierley J, Cho C, Wong RK (2013). Sequential phase I and II trials of stereotactic body radiotherapy for locally advanced hepatocellular carcinoma. J Clin Oncol.

[10] Ma S, Jiao B, Liu X, Yi H, Kong D, Gao L (2010). Approach to radiation therapy in hepatocellular carcinoma. Cancer Treat Rev.

[11] Chen SW, Lin LC, Kuo YC, Liang JA, Kuo CC, Chiou JF (2014). Phase 2 study of combined sorafenib and radiation therapy in patients with advanced hepatocellular carcinoma. Int J Radiat Oncol Biol Phys.

[12] Munoz-Schuffenegger P, Barry A, Atenafu EG, Kim J, Brierley J, Ringash J (2021). Stereotactic body radiation therapy for hepatocellular carcinoma with Macrovascular invasion. Radiother Oncol.

[13] Chang WI, Kim BH, Kim YJ, Yoon JH, Jung YJ, Chie EK (2022). Role of radiotherapy in Barcelona clinic liver cancer stage C hepatocellular carcinoma treated with sorafenib. J Gastroenterol Hepatol.

[14] Kim BK, Kim DY, Byun HK, Choi HJ, Beom SH, Lee HW (2020). Efficacy and safety of liver-directed concurrent chemoradiotherapy and sequential sorafenib for advanced hepatocellular carcinoma: a prospective phase 2 trial. Int J Radiat Oncol Biol Phys.

[15] Zhang H (2018). Might Sorafenib combined with radiotherapy be better option for treating hepatocellular carcinoma with portal vein tumour thrombosis?. Liver Int.

[16] Barry A, Knox JJ, Wei AC, Dawson LA (2016). Can stereotactic body radiotherapy effectively treat hepatocellular carcinoma?. J Clin Oncol.

[17] Keane FK, Hong TS, Zhu AX (2018). Evolving systemic therapy in hepatocellular carcinoma: current management and opportunities for integration with radiotherapy. Semin Radiat Oncol.

[18] Rim CH, Park S, Shin IS, Yoon WS (2021). Is the concurrent use of sorafenib and external radiotherapy feasible for advanced hepatocellular carcinoma? A meta-analysis. Cancers.

[19] Lu J, Zhao M, Arai Y, Zhong BY, Zhu HD, Qi XL (2021). Clinical practice of transarterial chemoembolization for hepatocellular carcinoma: consensus statement from an international expert panel of International Society of Multidisciplinary Interventional Oncology (ISMIO). Hepatobiliary Surg Nutr.

[20] Ikeda M, Shimizu S, Sato T, Morimoto M, Kojima Y, Inaba Y (2016). Sorafenib plus hepatic arterial infusion chemotherapy with cisplatin versus sorafenib for advanced hepatocellular carcinoma: randomized phase II trial. Ann Oncol.

[21] He M, Li Q, Zou R, Shen J, Fang W, Tan G (2019). Sorafenib plus hepatic arterial infusion of oxaliplatin, fluorouracil, and leucovorin vs sorafenib alone for hepatocellular carcinoma with portal vein invasion: a randomized clinical trial. JAMA Oncol.

[22] Zheng K, Zhu X, Fu S, Cao G, Li WQ, Xu L (2022). Sorafenib plus hepatic arterial infusion chemotherapy versus sorafenib for hepatocellular carcinoma with major portal vein tumor thrombosis: a randomized trial. Radiology.

[23] Lyu N, Wang X, Li JB, Lai JF, Chen QF, Li SL (2022). Arterial chemotherapy of oxaliplatin plus fluorouracil versus sorafenib in advanced hepatocellular carcinoma: a biomolecular exploratory, randomized, phase III trial (FOHAIC-1). J Clin Oncol.

[24] Ganten TM, Stauber RE, Schott E, Malfertheiner P, Buder R, Galle PR (2017). Sorafenib in patients with hepatocellular carcinoma-results of the observational INSIGHT study. Clin Cancer Res.

[25] Winters AC, Bedier F, Saab S (2020). Management of side effects of systemic therapies for hepatocellular carcinoma: guide for the hepatologist. Clin Liver Dis.

[26] Regmi P, Hu HJ, Lv TR, Paudyal A, Sah RB, Ma WJ (2021). Efficacy and safety of sorafenib plus hepatic arterial infusion chemotherapy for advanced hepatocellular carcinoma. Surg Oncol.

[27] Hu MD, Jia LH, Liu HB, Zhang KH, Guo GH (2016). Sorafenib in combination with transarterial chemoembolization for hepatocellular carcinoma: a meta-analysis. Eur Rev Med Pharmacol Sci.

[28] Chen J, He K, Han Y, Guo L, Su K, Wu Z (2022). Clinical efficacy and safety of external radiotherapy combined with sorafenib in the treatment of hepatocellular carcinoma: a systematic review and meta-analysis. Ann Hepatol.

[29] Tovoli F, Ielasi L, Casadei-Gardini A, Granito A, Foschi FG, Rovesti G (2019). Management of adverse events with tailored sorafenib dosing prolongs survival of hepatocellular carcinoma patients. J Hepatol.

[30] Liu S, Yu G, Wang Q, Li L, Liu Y, Du K (2021). CalliSpheres(®) microspheres drug-eluting bead transhepatic artery chemoembolization with or without sorafenib for the treatment of large liver cancer: a multi-center retrospective study. Am J Transl Res.

[31] Yuan J, Yin X, Tang B, Ma H, Zhang L, Li L (2019). Transarterial chemoembolization (TACE) combined with sorafenib in treatment of HBV background hepatocellular carcinoma with portal vein tumor thrombus: a propensity score matching study. Biomed Res Int.

[32] Lencioni R, Llovet JM, Han G, Tak WY, Yang J, Guglielmi A (2016). Sorafenib or placebo plus TACE with doxorubicin-eluting beads for intermediate stage HCC: the SPACE trial. J Hepatol.

[33] Kudo M, Ueshima K, Ikeda M, Torimura T, Tanabe N, Aikata H (2020). Randomised, multicentre prospective trial of transarterial chemoembolisation (TACE) plus sorafenib as compared with TACE alone in patients with hepatocellular carcinoma: TACTICS trial. Gut.

[34] Ren B, Wang W, Shen J, Li W, Ni C, Zhu X (2019). Transarterial chemoembolization (TACE) combined with sorafenib versus TACE alone for unresectable hepatocellular carcinoma: a propensity score matching study. J Cancer.

[35] Zhang YF, Wei W, Wang JH, Xu L, Jian PE, Xiao CZ (2016). Transarterial chemoembolization combined with sorafenib for the treatment of hepatocellular carcinoma with hepatic vein tumor thrombus. Onco Targets Ther.

[36] Liu KC, Hao YH, Lv WF, Jia WD, Ji CS, Zhou CZ (2020). Transarterial chemoembolization combined with sorafenib in patients with BCLC stage C hepatocellular carcinoma. Drug Des Dev Ther.

[37] Bai W, Wang YJ, Zhao Y, Qi XS, Yin ZX, He CY (2013). Sorafenib in combination with transarterial chemoembolization improves the survival of patients with unresectable hepatocellular carcinoma: a propensity score matching study. J Dig Dis.

[38] Koch C, Göller M, Schott E, Waidmann O, Op den Winkel M, Paprottka P (2021). Combination of sorafenib and transarterial chemoembolization in selected patients with advanced-stage hepatocellular carcinoma: a retrospective cohort study at three German liver centers. Cancers.

[39] Peng TR, Wu TW, Wu CC, Chang SY, Chan CY, Hsu CS (2022). Transarterial chemoembolization with or without sorafenib for hepatocellular carcinoma: a real-world propensity score-matched study. Tzu Chi Med J.

[40] Wan X, Zhai X, Yan Z, Yang P, Li J, Wu D (2016). Retrospective analysis of transarterial chemoembolization and sorafenib in Chinese patients with unresectable and recurrent hepatocellular carcinoma. Oncotarget.

[41] Kaibori M, Matsushima H, Ishizaki M, Kosaka H, Matsui K, Kariya S (2021). The impact of sorafenib in combination with transarterial chemoembolization on the outcomes of intermediate-stage hepatocellular carcinoma. Asian Pac J Cancer Prev.

[42] Hu H, Duan Z, Long X, Hertzanu Y, Shi H, Liu S (2014). Sorafenib combined with transarterial chemoembolization versus transarterial chemoembolization alone for advanced-stage hepatocellular carcinoma: a propensity score matching study. PLoS ONE.

[43] Meyer T, Fox R, Ma YT, Ross PJ, James MW, Sturgess R (2017). Sorafenib in combination with transarterial chemoembolisation in patients with unresectable hepatocellular carcinoma (TACE 2): a randomised placebo-controlled, double-blind, phase 3 trial. Lancet Gastroenterol Hepatol.

[44] Wu FX, Chen J, Bai T, Zhu SL, Yang TB, Qi LN (2017). The safety and efficacy of transarterial chemoembolization combined with sorafenib and sorafenib mono-therapy in patients with BCLC stage B/C hepatocellular carcinoma. BMC Cancer.

[45] Su D (2021). The transcatheter arterial chemoembolization combined with targeted nanoparticle delivering sorafenib system for the treatment of microvascular invasion of hepatocellular carcinoma. Bioengineered.

[46] Lee SW, Lee TY, Peng YC, Yang SS, Yeh HZ, Chang CS (2020). The therapeutic benefits of combined sorafenib and transarterial chemoembolization for advanced hepatocellular carcinoma. J Dig Dis.

[47] Zhang Y, Fan W, Wang Y, Lu L, Fu S, Yang J (2015). Sorafenib with and without transarterial chemoembolization for advanced hepatocellular carcinoma with main portal vein tumor thrombosis: a retrospective analysis. Oncologist.

[48] Park JW, Kim YJ, Kim DY, Bae SH, Paik SW, Lee YJ (2019). Sorafenib with or without concurrent transarterial chemoembolization in patients with advanced hepatocellular carcinoma: the phase III STAH trial. J Hepatol.

[49] Zhao S, Dou W, Fan Q, Hu J, Li H, Zhang X (2020). Identifying optimal candidates of transarterial chemoembolization (TACE) vs. sorafenib in patients with unresectable hepatocellular carcinoma. Ann Transl Med.

[50] Kirstein MM, Voigtländer T, Schweitzer N, Hinrichs JB, Marquardt J, Wörns MA (2018). Transarterial chemoembolization versus sorafenib in patients with hepatocellular carcinoma and extrahepatic disease. United Eur Gastroenterol J.

[51] Liu CM, Huang BS, Yen YH, Wang YM, Huang EY, Hsu HC (2021). Concurrent sorafenib and radiotherapy versus radiotherapy alone for locally advanced hepatocellular carcinoma: a propensity-matched analysis. J Hepatocell Carcinoma.

[52] Abulimiti M, Li Z, Wang H, Apiziaji P, Abulimiti Y, Tan Y (2021). Combination intensity-modulated radiotherapy and sorafenib improves outcomes in hepatocellular carcinoma with portal vein tumor thrombosis. J Oncol.

[53] Que J, Wu HC, Lin CH, Huang CI, Li LC, Ho CH (2020). Comparison of stereotactic body radiation therapy with and without sorafenib as treatment for hepatocellular carcinoma with portal vein tumor thrombosis. Medicine.

[54] Wada Y, Takami Y, Matsushima H, Tateishi M, Ryu T, Yoshitomi M (2018). The safety and efficacy of combination therapy of sorafenib and radiotherapy for advanced hepatocellular carcinoma: a retrospective study. Intern Med.

[55] Sun T, He J, Zhang S, Sun J, Zeng M, Zeng Z (2016). Simultaneous multitarget radiotherapy using helical tomotherapy and its combination with sorafenib for pulmonary metastases from hepatocellular carcinoma. Oncotarget.

[56] Bettinger D, Pinato DJ, Schultheiss M, Sharma R, Rimassa L, Pressiani T (2019). Stereotactic body radiation therapy as an alternative treatment for patients with hepatocellular carcinoma compared to sorafenib: a propensity score analysis. Liver Cancer.

[57] Nakazawa T, Hidaka H, Shibuya A, Okuwaki Y, Tanaka Y, Takada J (2014). Overall survival in response to sorafenib versus radiotherapy in unresectable hepatocellular carcinoma with major portal vein tumor thrombosis: propensity score analysis. BMC Gastroenterol.

[58] Liang RB, Zhao Y, He MK, Wen DS, Bu XY, Huang YX (2021). Hepatic arterial infusion chemotherapy of oxaliplatin, fluorouracil, and leucovorin with or without sorafenib as initial treatment for advanced hepatocellular carcinoma. Front Oncol.

[59] Miyaki D, Aikata H, Kan H, Fujino H, Urabe A, Masaki K (2013). Clinical outcome of sorafenib treatment in patients with advanced hepatocellular carcinoma refractory to hepatic arterial infusion chemotherapy. J Gastroenterol Hepatol.

[60] Kudo M, Ueshima K, Yokosuka O, Ogasawara S, Obi S, Izumi N (2018). Sorafenib plus low-dose cisplatin and fluorouracil hepatic arterial infusion chemotherapy versus sorafenib alone in patients with advanced hepatocellular carcinoma (SILIUS): a randomised, open label, phase 3 trial. Lancet Gastroenterol Hepatol.

[61] Kondo M, Morimoto M, Kobayashi S, Ohkawa S, Hidaka H, Nakazawa T (2019). Randomized, phase II trial of sequential hepatic arterial infusion chemotherapy and sorafenib versus sorafenib alone as initial therapy for advanced hepatocellular carcinoma: SCOOP-2 trial. BMC Cancer.

[62] Nagai H, Mukozu T, Ogino YU, Matsui D, Matsui T, Wakui N (2015). Sorafenib and hepatic arterial infusion chemotherapy for advanced hepatocellular carcinoma with portal vein tumor thrombus. Anticancer Res.

[63] Fukubayashi K, Tanaka M, Izumi K, Watanabe T, Fujie S, Kawasaki T (2015). Evaluation of sorafenib treatment and hepatic arterial infusion chemotherapy for advanced hepatocellular carcinoma: a comparative study using the propensity score matching method. Cancer Med.

[64] Zaizen Y, Nakano M, Fukumori K, Yano Y, Takaki K, Niizeki T (2021). Hepatic arterial infusion chemotherapy with cisplatin versus sorafenib for intrahepatic advanced hepatocellular carcinoma: a propensity score-matched analysis. Cancers.

[65] Therasse P, Arbuck SG, Eisenhauer EA, Wanders J, Kaplan RS, Rubinstein L (2000). New guidelines to evaluate the response to treatment in solid tumors. European Organization for Research and Treatment of Cancer, National Cancer Institute of the United States, National Cancer Institute of Canada. J Natl Cancer Inst.

[66] Lencioni R, Llovet JM (2010). Modified RECIST (mRECIST) assessment for hepatocellular carcinoma. Semin Liver Dis.

[67] Cox JD, Stetz J, Pajak TF (1995). Toxicity criteria of the radiation therapy oncology group (RTOG) and the European organization for research and treatment of cancer (EORTC). Int J Radiat Oncol Biol Phys.

[68] Choi SH, Seong J (2018). Strategic application of radiotherapy for hepatocellular carcinoma. Clin Mol Hepatol.

[69] Ohri N, Dawson LA, Krishnan S, Seong J, Cheng JC, Sarin SK (2016). Radiotherapy for hepatocellular carcinoma: new indications and directions for future study. J Natl Cancer Inst.

[70] Meyer T (2020). Stereotactic body radiotherapy for hepatocellular carcinoma—Still searching for a role. J Hepatol.

[71] Kim KH, Kim MS, Chang JS, Han KH, Kim DY, Seong J (2014). Therapeutic benefit of radiotherapy in huge (≥10 cm) unresectable hepatocellular carcinoma. Liver Int.

[72] Llovet JM, Ricci S, Mazzaferro V, Hilgard P, Gane E, Blanc JF (2008). Sorafenib in advanced hepatocellular carcinoma. N Engl J Med.

[73] Cheng AL, Kang YK, Chen Z, Tsao CJ, Qin S, Kim JS (2009). Efficacy and safety of sorafenib in patients in the Asia-Pacific region with advanced hepatocellular carcinoma: a phase III randomised, double-blind, placebo-controlled trial. Lancet Oncol.

[74] Bruix J, Raoul JL, Sherman M, Mazzaferro V, Bolondi L, Craxi A (2012). Efficacy and safety of sorafenib in patients with advanced hepatocellular carcinoma: subanalyses of a phase III trial. J Hepatol.

[75] Huang CY, Lin CS, Tai WT, Hsieh CY, Shiau CW, Cheng AL (2013). Sorafenib enhances radiation-induced apoptosis in hepatocellular carcinoma by inhibiting STAT3. Int J Radiat Oncol Biol Phys.

[76] Su K, Gu T, Xu K, Wang J, Liao H, Li X (2022). Gamma knife radiosurgery versus transcatheter arterial chemoembolization for hepatocellular carcinoma with portal vein tumor thrombus: a propensity score matching study. Hepatol Int.

[77] Zhou J, Sun H, Wang Z, Cong W, Wang J, Zeng M (2022). Guidelines for the Diagnosis and Treatment of Hepatocellular Carcinoma (2019 Edition). Liver Cancer.

[78] Yu W, Gu K, Yu Z, Yuan D, He M, Ma N (2013). Sorafenib potentiates irradiation effect in hepatocellular carcinoma in vitro and in vivo. Cancer Lett.

[79] Llovet JM, De Baere T, Kulik L, Haber PK, Greten TF, Meyer T (2021). Locoregional therapies in the era of molecular and immune treatments for hepatocellular carcinoma. Nat Rev Gastroenterol Hepatol.

[80] Díaz-González Á, Sapena V, Boix L, Brunet M, Torres F, LLarch N (2022). Pharmacokinetics and pharmacogenetics of sorafenib in patients with hepatocellular carcinoma: Implications for combination trials. Liver Int.

[81] Abeni E, Salvi A, Marchina E, Traversa M, Arici B, De Petro G (2017). Sorafenib induces variations of the DNA methylome in HA22T/VGH human hepatocellular carcinoma-derived cells. Int J Oncol.

[82] Chatterjee N, Bivona TG (2019). Polytherapy and targeted cancer drug resistance. Trends Cancer.

[83] Weng YS, Chiang IT, Tsai JJ, Liu YC, Hsu FT (2022). Lenvatinib synergistically promotes radiation therapy in hepatocellular carcinoma by inhibiting Src/STAT3/NF-κB-mediated epithelial-mesenchymal transition and metastasis. Int J Radiat Oncol Biol Phys.

[84] Shimose S, Iwamoto H, Tanaka M, Niizeki T, Shirono T, Noda Y (2021). Alternating lenvatinib and trans-arterial therapy prolongs overall survival in patients with inter-mediate stage hepatocellular carcinoma: a propensity score matching study. Cancers.

[85] Ando Y, Kawaoka T, Amioka K, Naruto K, Ogawa Y, Yoshikawa Y (2021). Efficacy and safety of lenvatinib-transcatheter arterial chemoembolization sequential therapy for patients with intermediate-stage hepatocellular carcinoma. Oncology.

[86] Chen S, Wu Z, Shi F, Mai Q, Wang L, Wang F (2022). Lenvatinib plus TACE with or without pembrolizumab for the treatment of initially unresectable hepatocellular carcinoma harbouring PD-L1 expression: a retrospective study. J Cancer Res Clin Oncol.

[87] Fu Z, Li X, Zhong J, Chen X, Cao K, Ding N (2021). Lenvatinib in combination with transarterial chemoembolization for treatment of unresectable hepatocellular carcinoma (uHCC): a retrospective controlled study. Hepatol Int.

[88] Ding X, Sun W, Li W, Shen Y, Guo X, Teng Y (2021). Transarterial chemoembolization plus lenvatinib versus transarterial chemoembolization plus sorafenib as first-line treatment for hepatocellular carcinoma with portal vein tumor thrombus: a prospective randomized study. Cancer.

[89] Peng Z, Fan W, Zhu B, Wang G, Sun J, Xiao C (2022). Lenvatinib combined with transarterial chemoembolization as first-line treatment for advanced hepatocellular carcinoma: a phase III, randomized clinical trial (LAUNCH). J Clin Oncol.

