# Supplementary material for: External radiotherapy combined with sorafenib has better efficacy in unresectable hepatocellular carcinoma: a systematic review and meta-analysis
Source: Clin Exp Med. 2022 Dec 10;23(5):1537–49. doi: 10.1007/s10238-022-00972-4 (PMC10460724; doi:10.1007/s10238-022-00972-4)
Supplement: Supplementary file 1 — Supplementary file1 (DOCX 33 kb) [file 10238_2022_972_MOESM1_ESM.docx]

| **Supplementary Table 1. Clinical results of included trials** | | | | | | | | | | | | | |  |
| --- | --- | --- | --- | --- | --- | --- | --- | --- | --- | --- | --- | --- | --- | --- |
| Author | Year | Arm | NO. | mOS | Survival rate | | | CR | PR | SD | PD | ORR | DCR |  |
|  |  |  |  |  | 1-year | 2-year | 3-year |  |  |  |  |  |  |  |
| Liu | 2021 | TACE | 48 | 12.70 | 0.55 | 0.21 | NA | NA | NA | NA | NA | 0.58 | 0.84 |  |
|  | 2021 | SOF+TACE | 42 | 18.60 | 0.77 | 0.36 | 0.11 | NA | NA | NA | NA | 0.80 | 0.98 |  |
| Yuan | 2019 | TACE | 138 | 7.00 | 0.41 | 0.15 | 0.09 | NA | NA | NA | NA | NA | NA |  |
|  | 2019 | SOF+TACE | 69 | 13.00 | 0.86 | 0.34 | 0.18 | NA | NA | NA | NA | NA | NA |  |
| Lencioni | 2016 | TACE | 153 | 9.70 | 0.77 | 0.54 | NA | 0.11 | 0.17 | 0.37 | 0.23 | 0.28 | 0.65 |  |
|  | 2016 | SOF+TACE | 154 | 9.60 | 0.69 | 0.57 | NA | 0.13 | 0.23 | 0.34 | 0.13 | 0.36 | 0.70 |  |
| Kudo | 2020 | TACE | 76 | NA | 0.77 | 0.65 | NA | 0.28 | 0.34 | 0.16 | 0.04 | 0.62 | 0.78 |  |
|  | 2020 | SOF+TACE | 80 | NA | 0.96 | 0.83 | NA | 0.29 | 0.43 | 0.13 | 0.03 | 0.72 | 0.84 |  |
| Ren | 2019 | TACE | 122 | 14.90 | 0.58 | 0.34 | 0.25 | NA | NA | NA | NA | NA | NA |  |
|  | 2019 | SOF+TACE | 61 | 29.00 | 0.74 | 0.53 | 0.40 | NA | NA | NA | NA | NA | NA |  |
| Zhang | 2016 | TACE | 60 | 6.10 | 0.15 | 0.12 | 0.06 | 0.02 | 0.18 | 0.23 | 0.43 | 0.20 | 0.43 |  |
|  | 2016 | SOF+TACE | 20 | 14.90 | 0.57 | 0.34 | 0.34 | NA | 0.50 | 0.30 | 0.80 | 0.50 | 0.80 |  |
| Liu | 2020 | TACE | 40 | 6.30 | 0.41 | 0.21 | 0.04 | 0.05 | 0.25 | 0.28 | 0.43 | 0.30 | 0.30 |  |
|  | 2020 | SOF+TACE | 35 | 13.60 | 0.71 | 0.46 | 0.13 | 0.09 | 0.34 | 0.40 | 0.17 | 0.43 | 0.43 |  |
| Bai | 2013 | TACE | 164 | 5.10 | 0.24 | NA | NA | 0.01 | 0.03 | 0.41 | 0.01 | 0.04 | 0.45 |  |
|  | 2013 | SOF+TACE | 82 | 7.50 | 0.32 | NA | NA | 0.01 | 0.10 | 0.49 | 0.01 | 0.11 | 0.60 |  |
| Koch | 2021 | SOF | 65 | 10.50 | 0.37 | 0.06 | NA | NA | 0.03 | 0.20 | 0.77 | 0.03 | 0.23 |  |
|  | 2021 | TACE | 54 | 16.50 | 0.56 | 0.13 | NA | NA | 0.09 | 0.29 | 0.62 | 0.09 | 0.38 |  |
|  | 2021 | SOF+TACE | 82 | 8.40 | 0.28 | 0.05 | NA | NA | 0.15 | 0.38 | 0.47 | 0.15 | 0.53 |  |
| Peng | 2022 | TACE | 112 | 5.20 | 0.12 | 0.05 | 0.02 | NA | NA | NA | NA | NA | NA |  |
|  | 2022 | Sof+TACE | 56 | 18.60 | 0.68 | 0.38 | 0.39 | NA | NA | NA | NA | NA | NA |  |
| Wan | 2016 | TACE | 245 | 13.97 | 0.55 | 0.34 | 0.22 | NA | NA | NA | NA | NA | NA |  |
|  | 2016 | Sof+TACE | 245 | 20.23 | 0.63 | 0.44 | 0.31 | NA | NA | NA | NA | NA | NA |  |
| Kaibori | 2021 | TACE | 29 | 15.40 | 0.78 | 0.43 | 0.33 | 0.01 | 0.10 | 0.55 | 0.34 | 0.11 | 0.66 |  |
|  | 2021 | Sof+TACE | 41 | 20.50 | 0.71 | 0.20 | 0.04 | 0.01 | 0.05 | 0.73 | 0.22 | 0.06 | 0.79 |  |
| Hu | 2014 | TACE | 164 | 4.90 | 0.15 | 0.02 | NA | NA | NA | NA | NA | NA | NA |  |
|  | 2014 | Sof+TACE | 82 | 7.00 | 0.25 | 0.13 | NA | NA | NA | NA | NA | NA | NA |  |
| Meyer | 2017 | TACE | 156 | 21.35 | 0.63 | 0.38 | 0.22 | 0.03 | 0.28 | 0.46 | 0.11 | 0.31 | 0.77 |  |
|  | 2017 | Sof+TACE | 157 | 22.50 | 0.69 | 0.45 | 0.24 | 0.03 | 0.33 | 0.49 | 0.10 | 0.36 | 0.85 |  |
| Wu | 2017 | SOF | 48 | 18.00 | 0.90 | 0.49 | 0.31 | 0.05 | 0.08 | 0.45 | 0.43 | 0.13 | 0.58 |  |
|  | 2017 | SOF+TACE | 56 | 22.00 | 0.88 | 0.60 | 0.39 | 0.06 | 0.12 | 0.38 | 0.44 | 0.18 | 0.56 |  |
| Su | 2021 | SOF | 24 | NA | NA | NA | NA | NA | NA | NA | NA | NA | 0.59 |  |
|  | 2021 | SOF+TACE | 18 | NA | NA | NA | NA | NA | NA | NA | NA | NA | 0.89 |  |
| Lee | 2020 | SOF | 65 | 6.00 | 0.28 | NA | NA | NA | NA | NA | NA | NA | NA |  |
|  | 2020 | SOF+TACE | 53 | 11.20 | 0.44 | NA | NA | NA | NA | NA | NA | NA | NA |  |
| Zhang | 2015 | SOF | 44 | 6.00 | 0.13 | NA | NA | NA | 0.09 | 0.48 | 0.16 | 0.09 | 0.57 |  |
|  | 2015 | SOF+TACE | 45 | 7.00 | 0.15 | NA | NA | NA | 0.21 | 0.35 | 0.23 | 0.21 | 0.56 |  |
| Park | 2018 | SOF | 169 | 10.80 | 0.44 | 0.22 | 0.21 | 0.01 | 0.06 | 0.42 | 0.36 | 0.07 | 0.49 |  |
|  | 2018 | SOF+TACE | 170 | 12.80 | 0.56 | 0.29 | 0.20 | 0.02 | 0.10 | 0.49 | 0.20 | 0.12 | 0.61 |  |
| Zhao | 2020 | SOF | 90 | 13.00 | 0.56 | 0.30 | 0.30 | NA | NA | NA | NA | NA | NA |  |
|  | 2020 | TACE | 233 | 19.20 | 0.68 | 0.43 | 0.41 | NA | NA | NA | NA | NA | NA |  |
| Kirstein | 2017 | SOF | 98 | 7.00 | NA | NA | NA | NA | NA | NA | NA | NA | NA |  |
|  | 2017 | TACE | 73 | 8.00 | NA | NA | NA | NA | NA | NA | NA | NA | NA |  |
| Liu | 2021 | RT | 73 | 9.60 | 0.37 | 0.26 | 0.14 | NA | NA | NA | NA | NA | NA |  |
|  | 2021 | SOF+RT | 73 | 9.90 | 0.38 | 0.23 | 0.21 | NA | NA | NA | NA | NA | NA |  |
| Abulimiti | 2021 | RT | 46 | 9.00 | 0.29 | 0.03 | 0.01 | 0.02 | 0.43 | 0.28 | 0.26 | 0.45 | 0.73 |  |
|  | 2021 | SOF+RT | 36 | 11.00 | 0.45 | 0.04 | 0.01 | 0.01 | 0.61 | 0.28 | 0.11 | 0.62 | 0.90 |  |
| Que | 2019 | RT | 36 | 7.00 | 0.33 | 0.11 | NA | 0.25 | 0.50 | 0.03 | 0.22 | 0.75 | 0.78 |  |
|  | 2019 | SOF+RT | 18 | 12.50 | 0.56 | 0.18 | NA | 0.33 | 0.44 | 0.11 | 0.11 | 0.77 | 0.88 |  |
| Sun | 2016 | RT | 18 | 29.60 | 1.00 | 0.94 | 0.50 | NA | NA | NA | NA | NA | NA |  |
|  | 2016 | SOF | 22 | 22.00 | 0.67 | 0.30 | 0.19 | NA | NA | NA | NA | NA | NA |  |
|  | 2016 | SOF+RT | 23 | 29.60 | 0.91 | 0.79 | 0.45 | NA | NA | NA | NA | NA | NA |  |
| Yoshiyuki | 2018 | RT | 47 | 12.10 | 0.48 | 0.12 | 0.01 | NA | 0.02 | 0.28 | 0.70 | 0.02 | 0.30 |  |
|  | 2018 | SOF+RT | 15 | 31.20 | 0.92 | 0.57 | 0.46 | 0.07 | 0.40 | 0.53 | 0.00 | 0.47 | 1.00 |  |
| Chang | 2022 | SOF | 330 | 9.50 | 0.43 | 0.14 | 0.09 | NA | NA | NA | NA | NA | NA |  |
|  | 2022 | SOF+RT | 68 | 15.70 | 0.59 | 0.33 | 0.21 | NA | NA | NA | NA | NA | NA |  |
| Bettinger | 2018 | SOF | 95 | 9.60 | 0.39 | 0.17 | 0.07 | NA | NA | NA | NA | NA | NA |  |
|  | 2018 | RT | 95 | 16.00 | 0.54 | 0.34 | 0.23 | NA | NA | NA | NA | NA | NA |  |
| Nakazawa | 2014 | SOF | 28 | 4.80 | 0.29 | 0.01 | 0.01 | NA | NA | NA | NA | NA | NA |  |
|  | 2014 | RT | 28 | 10.90 | 0.48 | 0.14 | 0.09 | NA | NA | NA | NA | NA | NA |  |
| Liang | 2021 | HAIC | 126 | 10.50 | 0.39 | 0.14 | 0.08 | 0.01 | 0.37 | 0.25 | 0.39 | 0.38 | 0.63 |  |
|  | 2021 | SOF+HAIC | 99 | 12.90 | 0.54 | 0.21 | 0.12 | 0.01 | 0.37 | 0.37 | 0.25 | 0.38 | 0.75 |  |
| Miyaki | 2019 | HAIC | 164 | 8.70 | 0.42 | 0.21 | 0.14 | 0.04 | 0.26 | 0.35 | 0.27 | 0.30 | 0.65 |  |
|  | 2019 | SOF+HAIC | 27 | 22.20 | 0.74 | 0.38 | 0.32 | 0.04 | 0.22 | 0.30 | 0.37 | 0.26 | 0.56 |  |
| He | 2015 | SOF | 20 | 7.90 | 0.23 | 0.11 | 0.02 | NA | NA | NA | NA | NA | NA |  |
|  | 2015 | SOF+HAIC | 18 | 10.70 | 0.42 | 0.36 | 0.36 | NA | NA | NA | NA | NA | NA |  |
| Kudo | 2019 | SOF | 122 | 7.10 | 0.22 | 0.13 | NA | 0.01 | 0.03 | 0.47 | 0.43 | 0.04 | 0.51 |  |
|  | 2019 | SOF+HAIC | 125 | 13.40 | 0.60 | 0.31 | NA | 0.01 | 0.41 | 0.34 | 0.20 | 0.42 | 0.76 |  |
| Kondo | 2018 | SOF | 103 | 11.50 | 0.46 | 0.22 | 0.12 | NA | NA | NA | NA | NA | NA |  |
|  | 2018 | SOF+HAIC | 102 | 11.80 | 0.47 | 0.23 | 0.12 | NA | NA | NA | NA | NA | NA |  |
| Zheng | 2019 | SOF | 35 | 15.20 | 0.58 | 0.24 | NA | 0.01 | 0.09 | 0.36 | 0.46 | 0.10 | 0.46 |  |
|  | 2019 | SOF+HAIC | 35 | 10.00 | 0.46 | 0.22 | NA | 0.01 | 0.14 | 0.31 | 0.46 | 0.15 | 0.46 |  |
| Ikeda | 2022 | SOF | 32 | 6.50 | 0.19 | 0.01 | 0.01 | 0.01 | 0.03 | 0.34 | 0.41 | 0.04 | 0.38 |  |
|  | 2022 | SOF+HAIC | 32 | 16.30 | 0.56 | 0.46 | 0.24 | 0.01 | 0.41 | 0.41 | 0.06 | 0.42 | 0.83 |  |
| Nagai | 2016 | HAIC | 42 | 8.70 | 0.34 | 0.13 | NA | NA | NA | NA | NA | NA | NA |  |
|  | 2016 | SOF+HAIC | 66 | 10.60 | 0.44 | 0.23 | 0.21 | NA | NA | NA | NA | NA | NA |  |
| Kotaro | 2015 | SOF | 72 | 12.50 | NA | NA | NA | 0.04 | 0.11 | 0.60 | 0.25 | 0.15 | 0.75 |  |
|  | 2015 | HAIC | 128 | 8.80 | NA | NA | NA | 0.04 | 0.23 | 0.41 | 0.32 | 0.27 | 0.68 |  |
| Lyu | 2018 | SOF | 232 | 7.00 | 0.07 | 0.01 | 0.01 | NA | NA | NA | NA | NA | NA |  |
|  | 2018 | HAIC | 180 | 14.50 | 0.66 | 0.22 | 0.05 | NA | NA | NA | NA | NA | NA |  |
| Zaizen | 2021 | SOF | 83 | 11.00 | 0.48 | 0.18 | 0.08 | NA | NA | NA | NA | NA | NA |  |
|  | 2021 | HAIC | 83 | 15.60 | 0.59 | 0.39 | 0.21 | NA | NA | NA | NA | NA | NA |  |
| Aoka | 2015 | SOF | 41 | 10.00 | 0.44 | 0.19 | 0.13 | 0.02 | 0.02 | 0.44 | 0.42 | 0.04 | 0.48 |  |
|  | 2015 | HAIC | 136 | 10.00 | 0.45 | 0.22 | 0.14 | 0.06 | 0.25 | 0.40 | 0.21 | 0.31 | 0.71 |  |
| Choi | 2018 | SOF | 29 | 7.20 | 0.19 | 0.18 | NA | NA | NA | NA | NA | NA | NA |  |
|  | 2018 | HAIC | 29 | 14.90 | 0.60 | 0.45 | NA | NA | NA | NA | NA | NA | NA |  |
| Kang | 2018 | SOF | 44 | 7.96 | 0.34 | 0.14 | 0.11 | NA | 0.02 | 0.36 | 0.61 | 0.02 | 0.38 |  |
|  | 2018 | HAIC | 95 | 12.80 | 0.24 | 0.10 | 0.05 | 0.02 | 0.21 | 0.34 | 0.43 | 0.23 | 0.57 |  |
| Ahn | 2020 | SOF | 35 | 6.40 | 0.01 | 0.01 | NA | NA | NA | NA | NA | NA | NA |  |
|  | 2020 | HAIC | 38 | 10.00 | 0.45 | 0.30 | NA | NA | NA | NA | NA | NA | NA |  |
| Moriguchi | 2017 | SOF | 14 | 4.30 | 0.08 | NA | NA | NA | NA | 0.29 | 0.50 | NA | 0.29 |  |
|  | 2017 | HAIC | 32 | 11.00 | 0.42 | 0.17 | NA | 0.03 | 0.28 | 0.25 | 0.38 | 0.31 | 0.56 |  |
| Song | 2015 | SOF | 60 | 5.50 | 0.14 | 0.05 | NA | NA | 0.13 | 0.32 | 0.55 | 0.13 | 0.45 |  |
|  | 2015 | HAIC | 50 | 7.10 | 0.34 | 0.16 | 0.13 | 0.02 | 0.22 | 0.66 | 0.10 | 0.24 | 0.90 |  |
| Abbreviations: mOS, median overall survival; CR, complete response; PR, partial response; SD, stable disease; PD, progressive disease; ORR, objective response rate; DCR, disease control rate. | | | | | | | | | | | | | |  |
|  |  |  |  |  |  |  |  |  |  |  |  |  |  |  |
